# Classification of Rhinoentomophthoromycosis into Atypical, Early, Intermediate, and Late Disease: A Proposal

**DOI:** 10.1371/journal.pntd.0003984

**Published:** 2015-10-01

**Authors:** Christian G. Blumentrath, Martin P. Grobusch, Pierre-Blaise Matsiégui, Friedrich Pahlke, Rella Zoleko-Manego, Solange Nzenze-Aféne, Barthélemy Mabicka, Maurizio Sanguinetti, Peter G. Kremsner, Frieder Schaumburg

**Affiliations:** 1 Institut für Tropenmedizin, Eberhard Karls Universität Tübingen and Deutsches Zentrum für Infektionsforschung, Tübingen, Germany; 2 Centre de Recherches Médicales de Lambaréné (CERMEL), Albert Schweitzer Hospital Lambaréné, Lambaréné, Gabon; 3 Centre de Recherche Médicale de la Ngounié, Fougamou, Gabon; 4 Center of Tropical Medicine and Travel Medicine, Department of Infectious Diseases, Division of Internal Medicine, Academic Medical Center, University of Amsterdam, Amsterdam, The Netherlands; 5 Evidat, Statistical Apps and Consulting, Sereetz, Germany; 6 Département de Parasitologie, Mycologie et Médicine Tropicale, Université des Sciences de la Santé, Libreville, Gabon; 7 Département d'Anatomie Pathologique et d`Histologie et Embryologie, Université des Sciences de la Santé, Libreville, Gabon; 8 Institute of Microbiology, Università Cattolica del Sacro Cuore, Rome, Italy; 9 Institute of Medical Microbiology, University Hospital Münster, Münster, Germany; University of Queensland, AUSTRALIA

## Abstract

**Background:**

Rhinoentomophthoromycosis, or rhino-facial conidiobolomycosis, is a rare, grossly disfiguring disease due to an infection with entomophthoralean fungi. We report a case of rhinoentomophthoromycosis from Gabon and suggest a staging system, which provides information on the prognosis and duration of antifungal therapy.

**Methods:**

We present a case of rhinoentomophthoromycosis including the histopathology, mycology, and course of disease. For the suggested staging system, all cases on confirmed rhinoentomophthoromycosis published in the literature without language restriction were eligible. Exclusion criteria were missing data on (i) duration of disease before correct diagnosis, (ii) outcome, and (iii) confirmation of entomophthoralean fungus infection by histopathology and/or mycology. We classified cases into atypical (orbital cellulitis, severe pain, fever, dissemination), early, intermediate, and late disease based on the duration of symptoms before diagnosis. The outcome was evaluated for each stage of disease.

**Findings:**

The literature search of the Medpilot database was conducted on January 13, 2014, (updated on January 18, 2015). The search yielded 8,333 results including 198 cases from 117 papers; of these, 145 met our inclusion criteria and were included in the final analysis. Median duration of treatment was 4, 3, 4, and 5 months in atypical, early, intermediate, and late disease, respectively. Cure rates were clearly associated with stage of disease and were 57%, 100%, 82%, and 43% in atypical, early, intermediate, and late disease, respectively.

**Conclusion:**

We suggest a clinical staging system that underlines the benefit of early case detection and may guide the duration of antifungal treatment. The scientific value of this classification is its capacity to structure and harmonize the clinical and research approach towards rhinoentomophthoromycosis.

## Introduction

Entomophthoromycoses, formally classified as a subgroup of phyco- and, later, zygomycoses, are rare, invasive fungal infections characterized by the formation of solid tumefactions [1–4]. Diseases due to entomophthoralean fungi are endemic in regions of tropical (rhino- and subcutaneous entomophthoromycosis) and arid (gastrointestinal entomophthoromycosis) climates [2,3].

Entomophthoralean fungi (*Conidiobolus coronatus*, *Conidiobolus incongruus*, *Conidiobolus lamprauges*, and *Basidiobolus ranarum*) live as saprophytes in soil and decaying plant matter [5]. Species were also isolated from surface water or faeces of insectivores [5,6]. Their ability to destroy insects coined the name of Entomophthorales [2]. These fungi cause infections in humans and mammals (e.g., horses, sheep, dogs, chimpanzees, and llamas) [7–11]. Potential sources of infection are contaminated soil, leaf litter, insects, and water [5,6].

**Fig 1:**
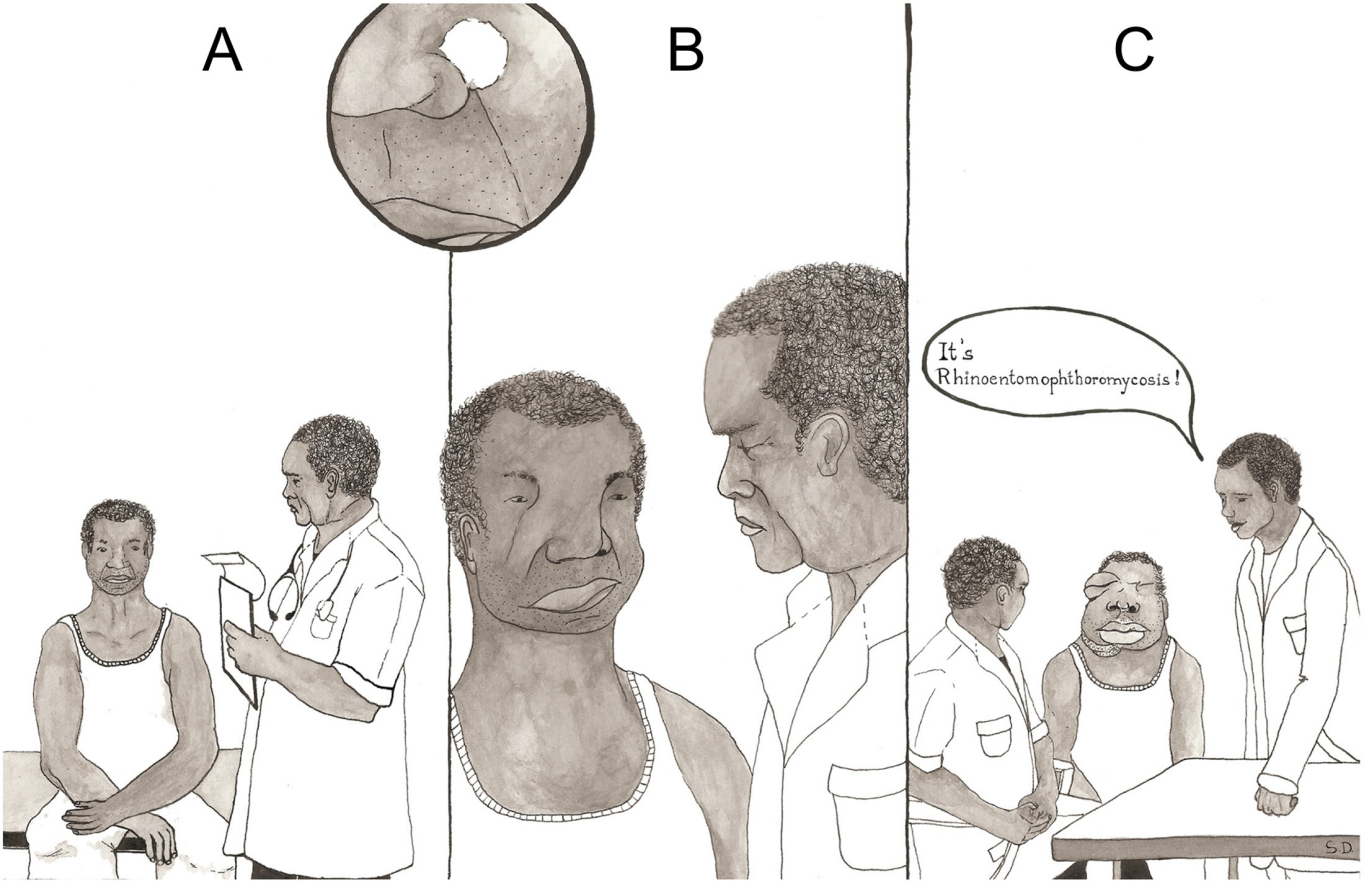
Typical course of rhinoentomophthoromycosis. (A) In early disease (cure rate: 100%) patients complain about rhinitis, intermittent epistaxis, nasal obstruction, or sinus pain. A nodule at the nostrils indicates invasion into the subcutaneous fat. Tissue invasion follows anatomical barriers causing facial tumefaction. (B) History of the disease of not more than 12 months is classified as intermediate disease (cure rate: 82%). (C) In late disease, patients present with facial deformity (cure rate: 43%). Patients with facial elephantiasis, as defined by Choon et al., might have an even poorer prognosis (cure rate: 37.5%) [2].

**Fig 2:**
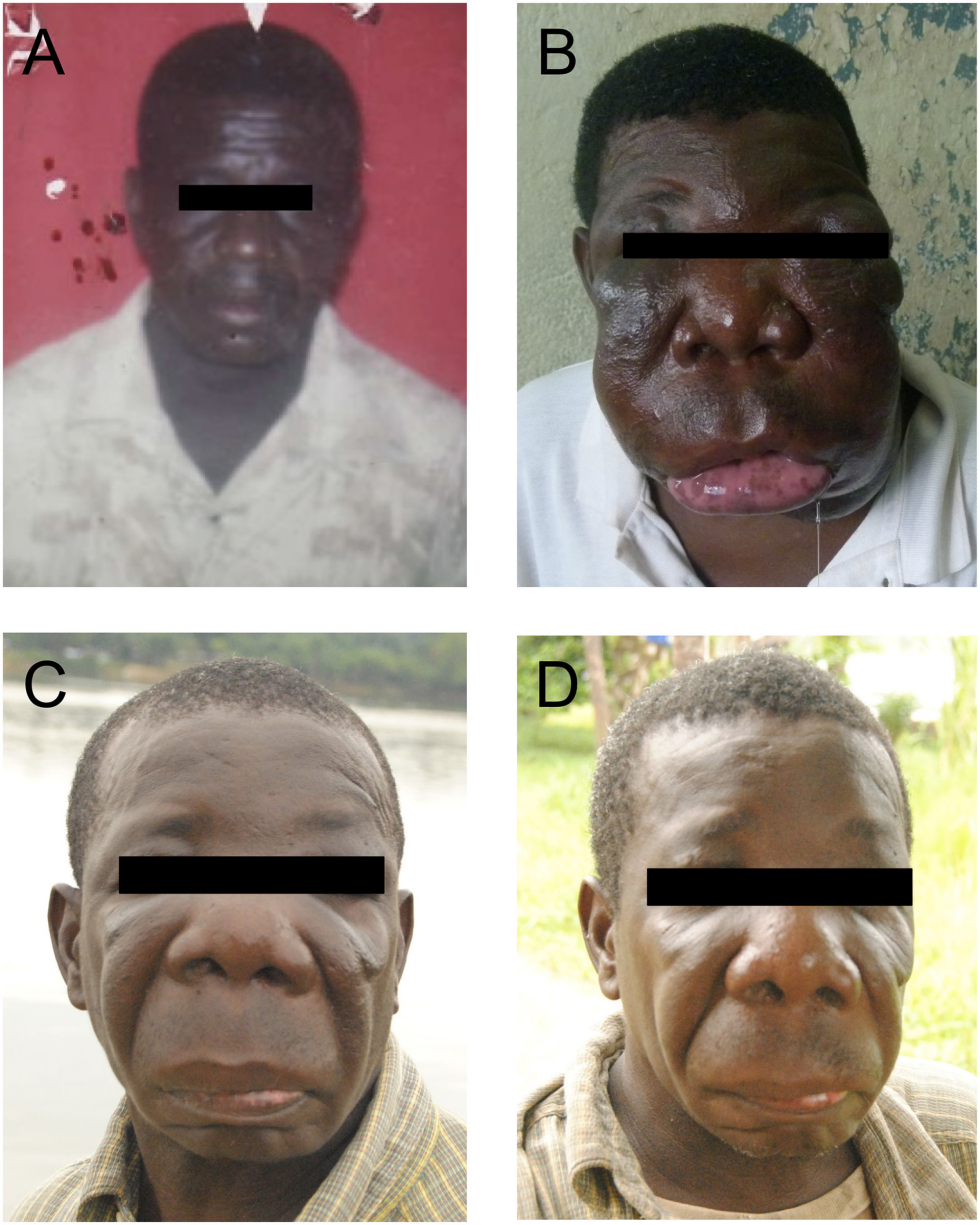
Portrait of the patient before, during and after treatment. (A) The patient is shown as a healthy young adult. (B) Before treatment (month 0). (C) After treatment with fluconazole and terbinafine (month 18). (D) After 14 months without therapy (month 32).

Humans suffering from rhinoentomophthoromycosis get infected by the attachment of conidia of *C*. *coronatus* to nasal/sinusoidal mucosa. Initially, the disease presents like sinusitis [2]. A nodule at the nostrils indicates expansion into the subcutaneous fat (Fig 1) [12,13]. The infection spreads within the subcutaneous fatty layers of the nasal bridge, eyelids, cheek, and upper lip [2,13]. Swellings are firm, indolent, and, initially, often reddened and warm, while later they are often itchy [2,12–15]. Mucosal swellings rarely affect laryngeal structures or cause dyspnoea [2]. Grotesque facial swelling is characteristic of late disease [2]. Ulcerations of skin or mucosa are uncommon; skin-adherent structures, eye motility, and vision usually remain unaffected; and bones, vessels, muscle, and lymph nodes are rarely involved. The course of the disease is usually benign (Figs 1 and 2) [2,14,15].

Choon et al. defined atypical rhinoentomophthoromycosis as a fungal infection of the facial region (e.g., orbit) other than the nose and maxillary sinus or in non-facial cutaneous sites [2]. In atypical disease, the fungus (*C*. *coronatus*, *C*. *inconguus*) can spread systemically (e.g., visceral organs) with poor prognosis [2].

**Fig 3:**
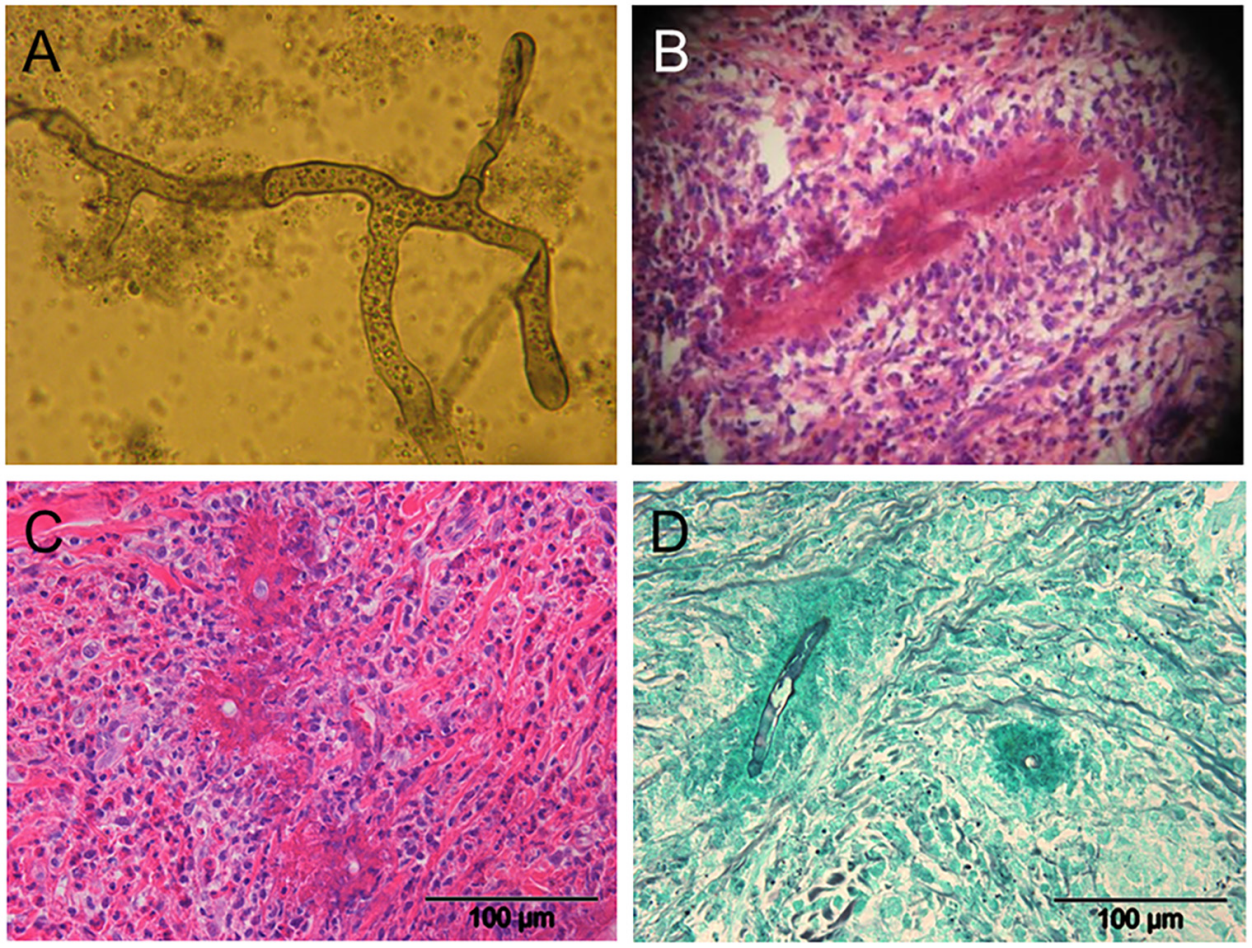
Histopathology. Histopathological picture of hyphae of Entomophthorales after KOH-staining (A), after hematoxylin eosin stain (HE-stain) (B and C), and after Grocott-Gomori's methenamine silver stain (GMS-stain) (D). The pauci-septated, right-angled branching fungal hyphae have an irregular diameter of 5–12 μm with drumstick-like distended ends (A). The “Splendore-Hoeppli phenomenon” is characterized by a peri-hyphal amorphous eosinophilic material (B). Fungal hyphae are visible in transversal (C) or longitudinal cuts (D).

Correct diagnosis of entomophthoromycoses is a challenge, as biopsies rarely reveal characteristic fungal hyphae in potassium hydroxide smears (Fig 3A). Hematoxylin eosin staining demonstrates an intense Splendore-Hoeppli-Phenomenon around unstained fungal hyphae and massive tissue infiltration by eosinophils; Gomori methamine silver and periodic acid Schiff coloration stain fungal cell walls (Fig 3B, 3C and 3D) [2,12,14,15]. Mycological cultures become positive in approximately 50% of cases [2,12,14,15]. Flat, waxy, white colonies grow within 3–10 days on Sabouraud agar (37°C), which become brownish over time [14]. Crown shaped, villous conidia are characteristic of *C*. *coronatus* [14,16,17]. Serologic, intracutaneous, and PCR tests are available in specialised laboratories [12,18].

Rhinoentomophthoromycosis is largely unknown, even in tropical regions. This lack of knowledge almost inevitably leads to a delay in establishing the correct diagnosis, and, consequently, to a poorer outcome. Therefore, we see a need to improve knowledge of this disease to guide diagnostic steps, prognosis of outcome, and antifungal therapy. However, a stage-adapted therapy and prognosis has not been developed so far.

Here we present the first case of a severe rhinoentomophthoromycosis from Gabon, in central Africa, and suggest a staging system that provides guidance for the prognosis of outcome and duration of antifungal treatment.

## Methods

### Case report

In April 2012, a 54-year-old male patient presented to the outpatient department of the “Centre de Recherche Médicale de la Ngounié,” Fougamou, Gabon. He complained of grotesque facial deformity (Fig 2), dysarthria, hypersalivation, and incapacity to eat properly.

In July 2009, he had a persisting rhinitis and recurrent epistaxis, while he was still working as a gardener. Six months later, a nodule occurred on his right nostril. Within three months, the nodule spread to the nasal bridge, causing reddish and hyperthermic swellings.

Physical examination revealed ligneous hard swellings of the whole face and left part of the neck. The upper and lower eyelids of both eyes were swollen, impairing his vision. No ulcerations of the skin or mucosa and no enlarged lymph nodes were noticed.

Laboratory analyses revealed an anaemia (haemoglobin 8.1 g/dl) and an absolute (2290/mm^3^) and relative (29%) eosinophilia, no leucocytosis. Microfilaria of *Loa Loa* were detected in venous EDTA blood (Citrate-Saponin acid method) and stool samples revealed an infection with *Ascaris lumbricoides* and *Trichuris trichiura*. We found no serologic evidence for hepatitis A, hepatitis B, syphilis, or HIV infection.

A rhinoentomophthoromycosis was suspected and a biopsy was taken (buccal region, 1 cm^3^) including the skin and indurated subcutaneous tissue. Histopathology revealed fungal hyphae suggestive for *Conidiobolus* sp. (Fig 3A, 3C and 3D) and the Splendore-Hoeppli phenomenon (Fig 3B). The histopathology did not show any evidence for tuberculosis, leprosy, or any malignancy. Fungal cultures showed no growth. DNA from clinical samples was extracted and amplified using fungus-specific primers [18]. A BLAST search using the sequence of the 676bp amplicon yielded a 100% match with sequences of *C*. *coronatus* (HQ602777.1, AJ345094.1, and AY997041.1).

Antifungal therapy (fluconazole 400 mg, terbinafine 250 mg, SID, po) was started in April 2012. After partial response during the first nine months of treatment, no further reductions were seen in the following three months. Dosages were therefore increased (to fluconazole 800mg, terbinafine 500mg, SID, po) and given for further six months. We observed no further improvement and stopped the treatment in September 2013 after 18 months in total. Helminth infections were treated with albendazole 400 mg (day 1, 3, and 9, po). Ferrous glycine sulfate (100 mg, BDS, po) was given for one month, and the haemoglobin level normalized (haemoglobin 13.7 g/dl). Serum transaminases (ASAT, ALAT) and creatinine were controlled every 30 days to monitor potential side effects. The laboratory parameters stayed within normal limits. Residual swellings remained after treatment. The patient is free of relapse after 14 months without antifungal therapy (Fig 2D).

### Case Review

Where possible, we considered the recommended items proposed by Preferred Reporting Items for Systematic Reviews and Meta-Analyses (PRISMA) group.

Literature search was performed on January 13, 2013, and included literature published until December 31, 2013. An update was performed on January 18, 2015, to add all articles published between January 1 and December 31, 2014, plus the case that we reported here.

We assessed the literature using the medpilot portal (www.medpilot.de), which accesses several electronic databases, including Current Contents, Catalogue of the U.S. National Library of Medicine (NLM), and MEDLINE (see website for complete list of databases). Search terms were “phycomycosis,” “zygomycosis,” “entomophthoromycosis,” “entomophthora,” “conidiobolus,” “basidiobolus,” “conidiobolomycosis,” “basidiobolomycosis,” “facial swelling,” and “rhinoentomophthoromycosis” (S1 Table). Titles and abstracts were screened for eligibility criteria. All cases that were diagnosed by the authors of the papers as an infection of nasopharyngeal and/or facial structures due to entomophthorales were eligible. The case definition included (i) rhinitis and/or intermittent epistaxis and/or sinus pain and/or tissue augmentation of nasopharyngeal and/or facial structures and (ii) confirmation of an entomophthoralean fungi infection by histopathology, culture, or PCR.

The reference lists of all eligible articles were screened for additional articles that were not detected using the search terms. All manuscripts were fully reviewed for initial symptoms, course of the disease, diagnostic tools, therapy, and outcome.

All eligible cases were reviewed for year of publication, country, continent, climate zone, age, sex, duration of disease before diagnosis, symptoms (i.e., coryza, epistaxis, nasal obstruction, facial elephantiasis), tissue invasion (i.e., nasal mucosa, nasal bridge, cheek, upper and lower lip, eyelids, orbit, lymph nodes, viscera), diagnostics for confirmation (i.e., smear microscopy, culture, histology, presence of the Splendore-Hoeppli phenomenon), antifungal treatment (i.e., agent, duration of therapy), and outcome (i.e., residual swellings, cure, sequelae, or death during follow-up). Cure was defined as being disease free after treatment with complete resolution of all clinical symptoms. Residual swellings were considered when the patients were disease free after treatment but showed persisting tissue augmentation.

Cases were classified into atypical, early, intermediate, and late disease.

**Fig 4:**
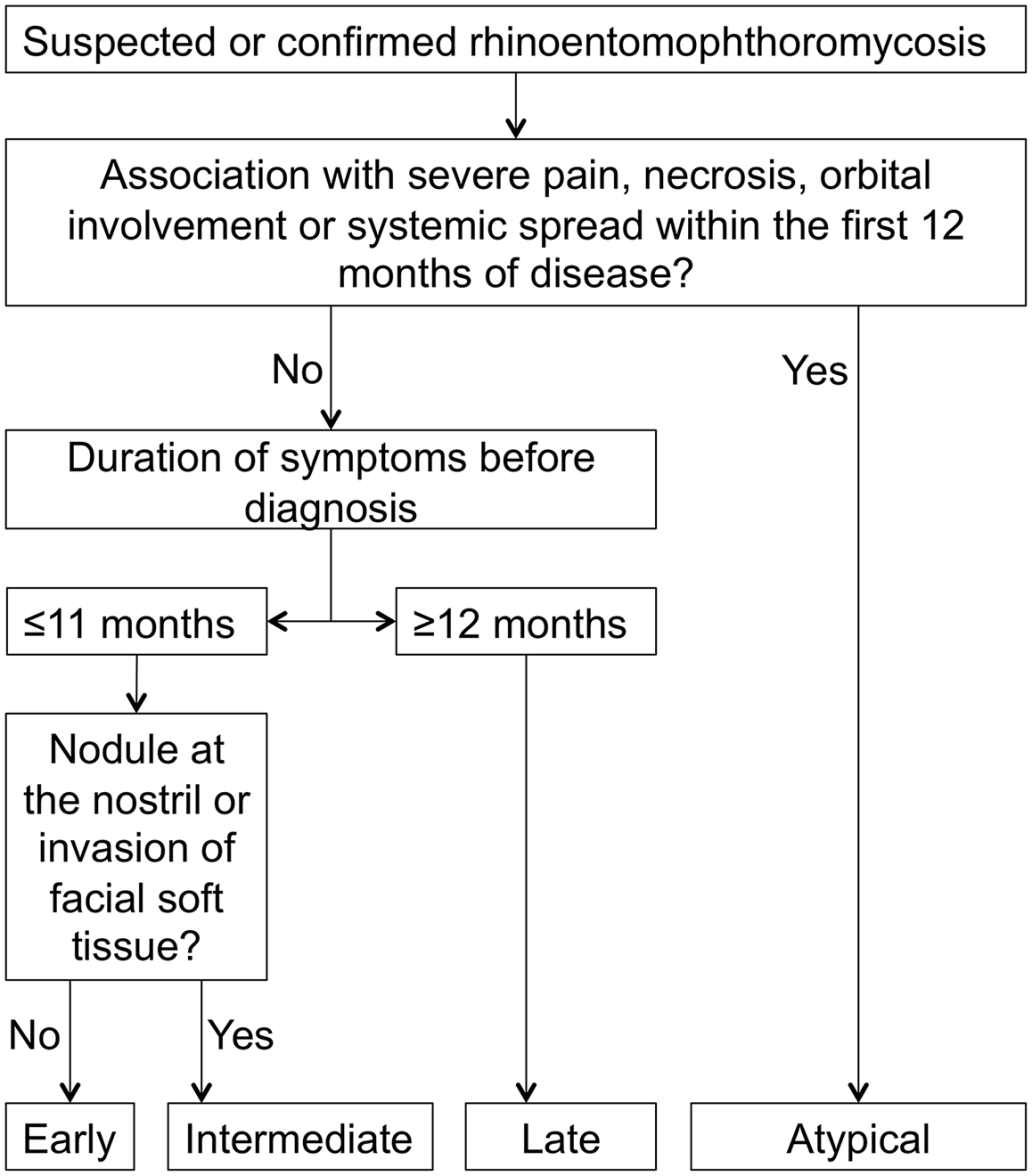
Algorithm for the staging of rhinoentomophthoromycosis. This algorithm is based on the clinical presentation of suspected/confirmed cases of rhinoentomophthoromycosis and the time since the onset of the first symptoms. The presence of a nodule at the nostril or invasion of facial tissue is the cornerstone to differentiate between early and intermediate disease.

Atypical disease was defined as ulcerations of the skin and/or invasion of the orbit, central nervous system, visceral organs, bones, muscles, and/or lymph nodes within 11 months after onset of disease (Fig 4).

We defined early disease as cases diagnosed before the occurrence of the characteristic nodule at the nostril (Fig 4).

Intermediate disease summarizes all cases diagnosed with facial swellings and a history of the disease ≤11 months (Fig 4).

All cases diagnosed with history of the disease ≥12 months were classified as late disease (Fig 4).

Exclusion criteria were (i) missing confirmation of entomophthoralean fungi infection by histopathology, culture, or PCR; (ii) missing information on duration of disease before diagnosis; and (iii) missing information on outcome.

Cases matching our case definition were analysed with IBM SPSS Statistics 22 (Armonk, New York, United States). Descriptive analyses were performed inter alia to check the distribution of parameters and Pearson’s correlation (r) was computed to determine the correlations of our review parameters. Homogeneity of review parameters among the different groups was tested using the Chi squared test for categorical variables and ANOVA for continuous variables.

Fischer’s exact test was performed with GraphPad PRISM 6 (www.graphpad.com, La Jolla, California, US) to determine the level of statistical significance (p<0.05) of the association of early, intermediate, and late disease with their corresponding cure rate. Two-tailed p values were calculated using the method of summing small p values. The significance level was set at 0.05. Additionally, odds ratio and relative risk were computed with MedCalc (www.medcalc.org, Ostend, Belgium) to assess the strength of the association of atypical, early, intermediate, and late disease with the corresponding cure rates. Normally distributed continuous variables were presented as mean values ± SD.

## Results

### Characteristics of the study population

**Fig 5:**
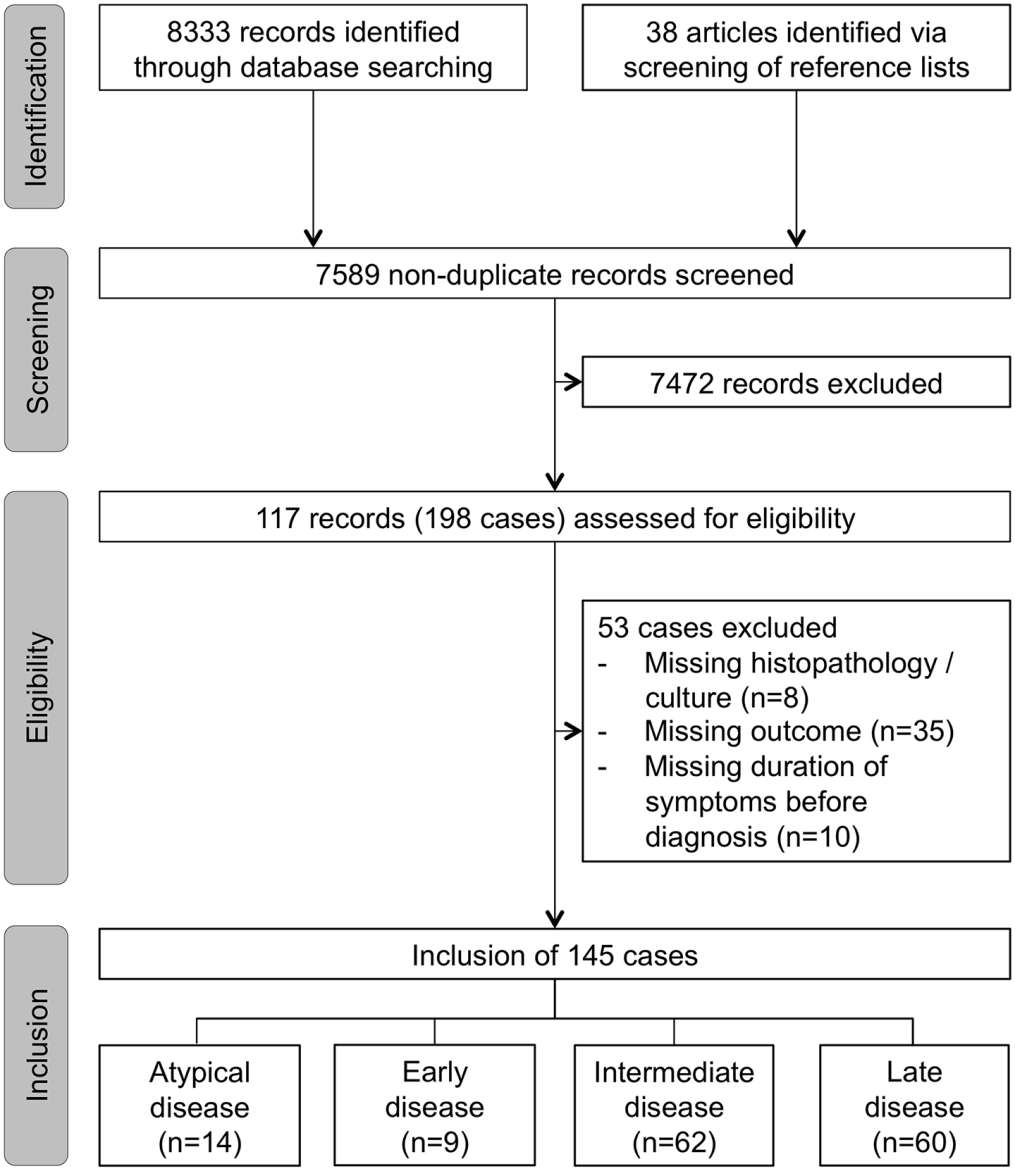
Flow chart of the selection of published cases on rhinoentomophthoromycosis. All cases assigned to staging analysis (n = 145) were included in the statistical analysis.

Search terms yielded 8,333 matches. By screening the reference lists of these manuscripts, we identified an additional 38 articles (Fig 5). In total, 198 cases reported in 117 articles met our case definition. Out of these cases, 53 cases were excluded due to missing essential information (Fig 5). A total of 145 cases provided data on duration of disease before diagnosis, outcome, and mycological or histopathological proof (Fig 5). In total, 145 cases were classified into early (n = 9), intermediate (n = 62), late (n = 60), and atypical (n = 14) disease (Table 1 and Fig 5). Of the 131 patients with atypical rhinoentomophthoromycosis, 95% (n = 124) were previously healthy. The concomitant diseases of the other seven patients were: helminthic infection (n = 3), HIV/tuberculosis coinfection (n = 1), tuberculosis (n = 1), diabetes mellitus (n = 1), and hypertension (n = 1). Atypical rhinoentomophthoromycosis presented as orbital cellulitis (100%, n = 14) associated with fever (71%, n = 10), severe sinus/orbital pain or headache (79%, n = 11), loss of vision (21% n = 3), and eye-movement restrictions (36% n = 5). Atypical disease patients had highly elevated infectious parameters (e.g., leukocytes, C-reactive protein, and erythrocyte sedimentation rate), which are uncommon in other stages. Systemic dissemination (i.e., lung and CNS) and comorbidities were more frequently found in atypical disease compared to other stages (86% vs. 2%, p<0.001 and 50% vs. 5%, p<0.001, respectively).

**Table 1:** Characteristics of the study population.

Characteristics		Total	Early	Intermediate	Late	Atypical course	p-value^a^
No of cases (n)		145	9	62	60	14	-
Age in years^b^	Median (range)	30 (0.01–79)	32 (19–79)	31 (5–65)	30 (11–72)	3 (0.01–78)	0.001
Sex	Male:female ratio, (n)	105:40	9:0	46:16	46:14	4:10	0.001
Pathogen^b^	*Conidiobolus coronatus*	61%	44.4%	61.3%	70%	29%	0.001
	Entomophthorales	23%	44.4%	29%	15%	14%	0.001
	*Conidiobolus* sp.	10%	11.1%	9.7%	10%	7%	0.74
	*Basidiobolus ranarum*	2%	0.0%	0.0%	3.3%	7%	0.001
	*Conidiobolus incongruus*	5%	0.0%	0.0%	1.7%	43%	0.001
No of drugs^b^	mean±SD	1.7±1.1	1.3±0.9	1.7±0.9	1.8±1.3	2.2±1.2	0.45
Surgical therapy		41%	100%	28%	43%	56%	0.001
Origin of cases	Africa	43%	11.1%	45.2%	52.5%	14%	0.001
	Asia	12%	44.4%	12.9%	3.4%	21%	0.001
	India	23%	22.2%	24.2%	20%	28%	0.6
	South/Middle America	18%	11.1%	16.1%	21.7%	14%	0.6
	Other	7%	11.2%	1.6%	2.4%	43%	0.001

^a^ Homogeneity was tested using one-way analysis of variance for continuous variables and Chi-square test for categorical variables.

^b^ The distribution of age (p = 0.86), mean number of antifungal drugs (p = 0.35), and all pathogens (p = 0.29) among early, intermediate, and late disease (excluding atypical disease) showed homogeneity.

Patients with atypical disease were younger (Table 1), more frequently immunosuppressed (29% vs. 0%, p<0.001), and more commonly female (76% versus 23%, p = 0.001). They had active tuberculosis more often (29% versus 3%, p<0.001), originated more likely from non-tropical regions (43% versus 3.1%, p<0.001), and had more frequent infections with *C*. *incongruus* (43% versus 0.8%, p<0.001) compared to patients with typical rhinoentomophthoromycosis (early, intermediate, and late disease).

As atypical disease differs from early, intermediate, and late disease in several aspects, we assessed the homogeneity of patient’s data only from early, intermediate, and late disease. These groups were comparable concerning age, distribution of all pathogens among the stages, and number of antifungal drugs (Table 1). Pooled data of early, intermediate, and late disease showed that duration of disease before diagnosis correlated inversely with cure (r = −0.34, p<0.001). Additionally, duration of disease before diagnosis correlated positively with residual swellings after treatment (r = 0.27, p = 0.02) and no response to treatment (r = 0.27, p = 0.03). Involvement of the lower lip (16%, n = 21) correlated negatively with cure (r = −0.34, p<0.001) and correlated positively with duration of disease before diagnosis (r = 0.38, p<0.001) and residual swellings after treatment (r = 0.32, p<0.001).

### Early disease

Early disease correlated with cure (r = 0.20, p = 0.03). In addition, there was an inverse correlation with residual swellings after treatment (r = -0.17, p = 0.05). In total, 100% (n = 9) were cured. According to Fischer’s exact test, the association of higher cure rates in early disease compared to pooled data from intermediate and late disease was statistically significant (Table 2). The odds ratio and relative risk revealed a lower risk of poor outcome in early disease, but did not reach statistical significance (Table 2). Mean duration of disease before diagnosis was 2.9±2.2 months (95% CI: 1.2–4.6 months). A mean number of 1.3±0.9 (95% CI: 0.5–2) different antifungal agents were used. Cases of early disease were treated for a median of three months (range 0.1–9 months). In all cases, surgical resection of infected sinus tissue was performed; one case was treated only by surgery. Cases were followed for a median time of 10 months (range 3–-66 months). A nodule at the nostrils indicates transition to intermediate disease as the fungi expand into the subcutaneous fatty layer.

**Table 2:** Comparison of outcomes in different groups.

Stage	Cure rate vs. cure rate of (pooled) other cases [%] ^a^ ^,^ ^b^ ^,^ ^c^	Risk assessment				Fischer´s exact test
		Odds ratio (95%CI)	p-value	Relative risk (95%CI)	p-value	p-value^e^
Early disease	100% versus 63%^a^	0.09 (0.01–1.6)	0.1	0.14 (0.01–2)	0.15	0.03
Intermediate disease	82% versus 43%^b^	0.17 (0.07–0.4)	<0.001	0.3 (0.18–0.56)	<0.001	<0.001
Late disease	43% versus 84%^c^	7.1 (3.1–16.2)	<0.001	3.66 (2.0–6.7)	<0.001	<0.001
Atypical course	57% versus 65%^d^	1.4 (0.5–4.3)	0.53	0.87 (0.5–1.4)	0.56	0.56

^a^ early disease versus intermediate and late disease

^b^ intermediate disease versus late disease

^c^ Late disease versus early and intermediate disease

^d^ atypical versus early, intermediate and late disease

^e^ Calculation was done using the method of summing small p values

### Intermediate disease

Intermediate disease correlated with cure after treatment (r = 0.33, p<0.001). However, cure rates were lower compared to early disease (82%, n = 51). Fischer’s exact test showed that the cure rate was significantly higher in intermediate compared to late disease (Table 2). Odds ratio and relative risk revealed a significantly better prognosis for cases of intermediate compared to late disease (Table 2). Furthermore, there was a negative correlation between intermediate disease and no response to treatment (r = -0.24, p = 0.05) and residual swellings after treatment (r = –0.24, p = 0.07). Mean duration of disease before diagnosis was 5±2.5 months (95% CI: 4.4–5.7 months). Surgery was performed in 28% (n = 17) of the cases. This included removal of infected sinus tissue (n = 13), partial removal of tumour masses and reconstructive surgery (n = 1), and reconstructive surgery alone (n = 3). In one case, surgery was the only treatment. The median treatment duration was four months (range: 0.1–48 months). A mean number of 1.7±0.9 (95% CI: 1.4–1.9) different antifungal agents were used (Table 1). The median follow-up period was 5 months (range: 0.3–72 months).

### Late disease

Late disease correlated inversely with cure (r = -0.43, p<0.001). Additionally, late disease correlated with residual swellings after treatment (r = 0.32, p<0.001) and no response to treatment (r = 0.28, p = 0.001). In total, only 43% (n = 26) of patients in this stage were cured. According to Fischer’s exact test, late disease was significantly associated with lower cure rates compared to pooled data from early and intermediate disease (Table 2). The calculation of odds ratio and relative risk demonstrated significantly higher risks of poor outcome results in cases from late disease compared to pooled data from early and intermediate disease (Table 2). Mean duration of facial tumefaction before diagnosis was 35±33 months (95% CI: 26–44 months). A mean number of 1.8±1.2 (95% CI: 1.5–2.2) different antifungal agents was used. Surgery was performed in 43% of the cases (n = 26). Of these, one case was surgically treated only (multiple step excision of tumour masses). Other surgical interventions were resection of infected sinus tissue (n = 7), excision of other infected tissue (n = 9), and reconstructive surgery after antifungal treatment (n = 10). Late disease cases were treated for a median of five months (range: 0.1–41 months, Table 1). The median follow-up period was eight months (range: 0.8–180 months).

### Atypical disease

Atypical disease (n = 14) did not correlate with cure after treatment, response to treatment, or cure with sequelae. No difference of cure rates was detected when comparing patients with atypical disease and patients with typical rhinoentomophthoromycosis (early, intermediate, and late disease in [Table 2]). In contrast, the lethality rate was significantly higher in atypical (43%, n = 6) compared to pooled data of early, intermediate, and late disease (0.7%, n = 1, p<0.001, OR = 14.8, 95%CI: 7.1–30.9). The mean duration of symptoms before diagnosis was 1.6±1.3 months (95% CI: 0.6–2.6 months). A mean number of 2.2±1.2 (95%CI: 1.3–3.2) antifungal drugs were used to treat atypical disease. Surgery was performed in 56% (n = 8). Out of these eight cases, surgical interventions included resection of infected sinus tissue alone (n = 2), the latter plus orbital decompression (n = 4), and orbital decompression alone (n = 2). Patients additionally receiving a hyperbaric oxygen therapy (n = 4) compared to patients who received antifungal treatment/surgery only (n = 10) had lower lethality rates (0% vs. 80%, p = 0.01). Hyperbaric oxygen therapy was not used in patients with other stages of disease. Atypical disease cases were treated for a median of four months (range: 0.3–11 months). The median follow up period was six months (range: 0.6–60 months).

### Treatment

Saturated potassium iodide solution (ssKI; 3x3 drops up to 3x40 drops per day) was used in 54% (n = 79) of the cases. Use of ssKI correlated very weakly with cure (r = 0.21, p = 0.02). Patients also received amphotericin B (32%, n = 46), ketoconazole (20%, n = 29), sulfamethoxazole/trimethoprim (15%, n = 21), itraconazole (16%, n = 23), fluconazole (15%, n = 21), miconazole (2%, n = 3), terbinafine (3%, n = 4), and 5-fluorocytosin (4%, n = 6). Two patients were treated with voriconazole and both were disease free after treatment. All antifungal agents were used equally among the different groups of early, intermediate, and late disease. The baseline drug in atypical disease was amphotericin B (n = 12), mostly used in combination with other antifungal compounds (n = 8), for instance, amphotericin B/5-fluorocytosin (n = 3), amphotericinB/ssKI (n = 2), amphotericin B/itraconazole (n = 2), and amphotericin B/ sulfamethoxazole/trimethoprim (n = 1).

Surgery was performed in 41% (n = 59) of all cases. Surgical interventions were excision of infected tissue (n = 37), reconstructive surgery after antifungal medication (n = 10), excision of infected tissue plus reconstructive surgery after antifungal medication (n = 10), excision of infected tissue plus orbital decompression (n = 4), and orbital decompression alone (n = 4). Surgery did not correlate with cure (r = −0.08, p = 0.30). We additionally compared cure rates of cases treated surgically versus cases without surgical interventions on group-levels (overall, in atypical, intermediate, and late disease) using Fischer’s exact test. There was no benefit for patients treated surgically in any of the groups (overall, p = 0.63; atypical disease, p = 0.57; intermediate disease, p = 0.73; and late disease, p = 0.31). The treatment of three typical disease cases was based on surgical interventions without any antifungal medication.

The overall cure rate was 63% (n = 92), partial cure with sequelae was achieved in 27% (n = 38), and 6% (n = 8) had no response to treatment. One typical disease patient and 43% (n = 6) of the atypical disease cases died during follow up (bacterial pneumonia in typical disease and sepsis or multi-organ failure in atypical disease [n = 6]), while three typical patients showed recurrence after initial cure.

## Discussion

Our study is capitalizing on the analyses of outcome determinants of rhinoentomophthoromycosis as provided by Choon et al. [2]. They identified visceral involvement, presence of comorbidities, an infection by *C*. *incongruus* or *Conidiobolus lamprauges*, and female sex as predictors of poorer outcome [2]. In addition, they defined typical and atypical conidiobolomycosis [2].

Here, we propose a staging system that classifies rhinoentomophthoromycosis into atypical, early, intermediate, and late disease, which provides information on the prognosis and duration of antifungal treatment (Table 2).

In contrast to our approach, Isa-Isa et al. applied a classification of rhinoentomophthoromycosis that is based on tissue invasion [12]. In phase I, the disease is limited to mucosal involvement without facial swellings. Phase II includes all cases that show facial swellings. In phase III, muscles, bones, and viscera are involved. This approach seems to be based on the assumption that the disease inevitably invades muscles, bones, and viscera. In rhinoentomophthoromycosis, phase III is a rarity and should be seen as a complication in late or atypical disease [2,14]. In conclusion, this classification has only limited implications on therapeutic strategies [12].

Unlike the slow progression of early, intermediate, and late disease, atypical disease is characterized by acute symptoms, for instance, severe sinus/facial pain, eye-movement restriction, loss of vision, orbital cellulitis, or rapid progression with involvement of cerebral structures or other internal organs. In general, atypical disease seems to affect mostly females and patients with insufficient immunocompetence (e.g., young patients, immunosuppression). This is supported by our finding that patients with an active tuberculosis were at risk for atypical disease as a *Mycobacterium tuberculosis* infection can reduce the host resistance to invasive fungal pathogens [19]. In immunosuppressed patients, atypical disease mimics the clinical and histopathological picture of mucormycosis as the “Splendore-Hoeppli phenomenon” and tissue infiltration by eosinophils are frequently absent while necrosis and vessel infiltration occurs [2,14,15,20–22]. *C*. *incongruus* was isolated in 40% of atypical cases. The geographic distribution of entomophthoralean fungi and their corresponding clinical manifestations (chronic forms in tropical climate versus acute forms in non-tropical or arid climates) suggest a different pathogenic potential of different species or a differing virulence of different lineages of the same species (Table 1). On the other hand, the higher prevalence of atypical and gastrointestinal entomophthoromycoses in non-tropical climates might not point towards differences in virulence of Entomophthorales. It could also be due to improved medical services in non-tropical countries that have the capacity to detect even gastrointestinal entomophthoromycoses, which might remain undetected in resource-limited tropical countries.

Frequently, the diagnosis of rhinoentomophthoromycosis is based on clinical signs and the patient’s history (Fig 1). Where possible, a laboratory confirmation should be sought. The gold standard is microscopy of KOH-smears (Fig 3), culture on Sabouraud agar, and histopathology [2,5,6]. Granulomatous inflammation (neutrophils, eosinophils), fibrosis, granulation tissue, and the “Splendore Hoeppli” phenomenon is frequently found in tissues infected with Entomophthorales (Fig 3) [23]. Hyphae are thick, right-angled, and pauci-septated with drumstick-like distended ends (Fig 3). Recently, PCR techniques from native and formalin-embedded tissues were developed [16,18,24].

There is a lack of treatment guidelines for rhinoentomophthoromycosis. Adjuvant surgery after starting antifungal treatment could be an option in early disease as resection of infected tissue might contribute to improved outcome. In intermediate and late disease, surgery should be restricted to interventions in life-threatening events and reconstructive surgery after definitive residual cure [2,13]. Orally administered antifungal agents (e.g., azoles, ssKI, terbinafine, sulfamethoxazole/trimethoprim) have been shown to be effective in typical disease [2].

In atypical disease, some of the surgical interventions (e.g., orbital decompression) are unavoidable. The course of the disease often requires treatment in the intensive care unit. Hence, therapy is an interdisciplinary concept that involves infectious disease specialists, ophthalmologists, otolaryngologists, and intensive care specialists. Hyperbaric oxygen therapy significantly reduces the lethality rate of atypical disease.

Amphotericin B is the baseline drug in atypical disease. It is only available as an IV drug, which requires hospitalization for administration. Second, the huge number of side effects disqualify this drug in a setting where monitoring of renal parameters and electrolytes cannot be guaranteed. Based on our review of 198 cases, we suggest the use of the maximum-approved dosage of the selected drug, which is well tolerated by the patient. In low- and middle-income countries, ssKI, fluconazole, and sulfamethoxazole/trimethoprim might be considered as first line drugs as they are cheap and effective alternatives, at least in typical disease [1].

A combination of antifungal compounds (e.g., itraconazole/terbinafine, ssKI/sulfamethoxazole/trimethoprim, and fluconazole/itraconazole), not yet widely established and recommended, was given preference in treatment of rhinoentomophthoromycosis in recent studies [2,16,25].

Antifungal resistance patterns of entomophthoralean fungi vary among different species and among lineages of the same species [2]. In vitro susceptibility testing was not predictive for outcome [2]. Nevertheless, susceptibility testing is recommended because of multiple drug resistances discovered in vitro and may be helpful in case of suspected resistances [2,25,26]. However, access to susceptibility testing might be limited in resource restricted settings. In addition, to the best of our knowledge, standardized antifungal susceptibility testing with species related break points of minimal inhibitory concentration are not available for Entomophthorales.

In general, intermediate and late diseases require long-term treatment and, despite clinical cure, fungal hyphae can be found even after more than eight months of treatment [27,28]. Our suggested duration of treatment may serve as an orientation for minimum duration (Table 3). Differentiation between persistent fungal infection and cure with sequelae (e.g., due to lymphedema, excessive tissue) is a challenge when evaluating the outcome. Drug changes during therapy should be considered in case of suspected resistances or adverse events [27,28]. Weekly photo-documentation is helpful to estimate the response to treatment. Involving our suggested treatment durations, this strategy may reveal resistances, cure with sequelae, and avoid excessive treatment durations in typical course. In case of suspected resistances or cure with sequelae, resampling should be considered once the stage adapted suggested treatment duration is achieved.

**Table 3:** Suggested staging system of rhinoentomophthoromycosis.

	Stage of disease			
	Atypical	Early	Intermediate	Late
Definition	Aggressive form of the disease associated with immunosuppression	The infection is limited to the nasopharygeal tissue. A characteristic nodule indicates transition to intermediate disease.	Duration of symptoms <12 months	Duration of symptoms ≥12 months
Symptoms	Orbital cellulitis, severe sinus or orbital pain, epiphora, eye-movement restriction, pulmonary and cerebral dissemination	Rhinitis, epistaxis, nasal obstruction, sinus pain, tumefaction of sinus mucosa, nodule at nostrils	Painless tumefaction of nasal bridge, upper lip, cheek, eyelids, forehead. Warmth, rubor, fever, and malaise may be present.	Painless, sometimes itchy, hard tumefaction of nose, cheek, upper and lower lips, forehead, eyelids; usually no rubor, warmth. or ulceration; facial elephantiasis
Median duration since onset of symptoms (range)	2 months (0.3–4 months)	3 months (1–8 months)^a^	5 months (1–11 months)^a^	24 months (12–180 months)
Diagnostics	CT- or MR-scans reveal tissue invasion; serological tests in specialised laboratories; species differentiation by KOH-smears, culture, and PCR from biopsies of infected tissue. Histopathology reveals the indistinctive findings of entomophthoromycosis. In atypical course, infectious parameters are highly elevated and histopathological findings are similar to mucormycosis.			
Cure rate	57%^b^	100%	82%	43%
Recommended minimum duration of antifungal medication	4 months	3 months	4 months	5 months
Suggested treatment	Surgical resection of infected tissue, antifungal therapy, ideally based on susceptibility testing. Amphotericin B and combination of antifungal compounds; hyperbaric oxygen therapy.	Surgical resection of infected sinus tissue; additional oral antifungal therapy, ideally based on susceptibility testing (ssKI, azoles, terbinafine, trimethoprim/sulfamethoxazole or combinations).	No initial surgery. Oral antifungal therapy, ideally based on susceptibility testing (ssKI, azoles, trimethoprim/sulfamethoxazole, terbinafine, or combinations); reconstructive surgery in case of sequelae.	No initial surgery. Oral antifungal therapy, ideally based on susceptibility testing (ssKI, azoles, terbinafine, trimethoprim/sulfamethoxazole or combinations); reconstructive surgery in case of sequelae.

^a^ The duration of disease can overlap between early and intermediate disease. See Fig 4 for the definite association of a case to the corresponding stage.

^b^ Cure rates in atypical disease could be misleading. The lethality rate (43%) might be more conclusive.

The advantage of our proposed staging system is that physicians now have assistance to guide the treatment and the prognosis of the different stages of rhinoentomophthoromycosis. Our classification might also be used for systematic records of cases with rhinoentomophthoromycosis in disease registries of neglected diseases. The simple staging system might also be used for educational campaigns in endemic regions.

Although this staging system was developed based on the best available case reports and case series, some limitations need to be addressed. First, rhinoentomophthoromycosis is probably underdiagnosed and underreported. Cases were not analysed if they were not detected by our search strategy. Second, even if the correlations were statistically significant, they were only weak. However, outcome analyses (odds ratio, relative risk, and Fischer’s exact test) support our results. Third, reporting bias, reporting heterogeneity, and missing data might have been affecting our results as well. Fourth, the data heterogeneity of treatment regimens concerning drugs, dosages, and combinations did not allow for a meaningful analysis. Fifth, surgery might be a potential confounder of better outcome in early disease. We were not able to address this limitation adequately as all early cases underwent surgery. However, we rank the impact of surgery as a confounder of our analyses as low because surgery did not predict better outcome in a meta-analysis [2]. This is in line with our observations of a non-detectable impact of surgery on the outcome of the disease.

## Conclusion

The classification of rhinoentomophthoromycosis into early, intermediate, late, and atypical disease provides information on the prognosis of infection and duration of antifungal therapy. The analysis highlights the benefit of an early case detection.

**Key Learning Points**

- Entomophthoromycoses are rare invasive fungal infections, characterized by the formation of solid tumours of the affected organs/region with a unique histopathology.
- Rhinoentomophthoromycosis affects the nasal and facial tissue and can be invasive to bones and the nervous system (atypical rhinoentomophthoromycosis).
- We suggest a classification of rhinoentomophthoromycosis into atypical, early, intermediate, and late disease that provides a stage dependent orientation on therapy and prognosis.
- Education campaigns in endemic countries should address the major issues of (rhino-)entomophthoromycosis.

**Top Five Papers**

1. Ribes JA, Vanover-Sams CL, Baker DJ. Zygomycetes in Human Disease. Clin Microbiol Infect. 2000; 13: 236–301.
2. Prabhu RM, Patel R. Mucormycosis and entomophthoramycosis: a review of the clinical manifestations, diagnosis and treatment. Clin Microbiol Infect. 2004; 10: 31–47.
3. Choon SE, Kang J, Neafie RC, Ragsdale B, Klassen-Fischer M, Carlson JA. Conidiobolomycosis in a young Malaysian woman showing chronic localized fibrosing leukocytoclastic vasculitis: a case report and meta-analysis focusing on clinicopathologic and therapeutic correlations with outcome. Am J Dermatopathol. 2012; 34: 511–522.
4. Holenarasipur R, Vikram JD, Smilack JA, Leighton M, Crowell D, De Petris G. Emergence of Gastrointestinal Basidiobolomycosis in the United States, With a Review of Worldwide Cases; Clin Infect. Dis. 2012; 54(12): 1685–169
5. Kombaté K, Saka B, Mouhari-Toure A, Akakpo S, Djadou KE, Darré T, et al. Basidiobolomycosis: a review; Med. Sante Trop. 2012; 22(2): 145–52

## Ethics Statement

The patient in this manuscript has given written informed consent to publication of his case details.

## Supporting Information

S1 Table Search strategies. (DOCX)
