# Supplementary material for: Classification of Rhinoentomophthoromycosis into Atypical, Early, Intermediate, and Late Disease: A Proposal
Source: PLoS Negl Trop Dis. 2015 Oct 1;9(10):e0003984. doi: 10.1371/journal.pntd.0003984 (PMC4591341; doi:10.1371/journal.pntd.0003984)
Supplement: S1 Table — (DOCX) [file pntd.0003984.s001.docx]

**Supplementary Material I – Search strategies**

Database: Medpilot (advanced search; open search), all records published until December 2013

Date of search: 13^th^ January 2014

| # | Search | Results |
| --- | --- | --- |
| 1 | exp basidiobolomycosis/ | 122 |
| 2 | exp basidiobolus/ | 171 |
| 3 | exp conidiobolomycosis/ | 25 |
| 4 | exp conidiobolus/ | 187 |
| 5 | exp entomophthoromycosis/ | 103 |
| 6 | exp entomophthora/ | 228 |
| 7 | exp facial swelling/ | 5165 |
| 8 | exp phycomycosis/ | 346 |
| 9 | exp rhinoentomophthoromycosis/ | 34 |
| 10 | exp zygomycosis/ | 1394 |
| 11 | Screening of reference lists of records identified in searches #1-#10 | 38 |

Database: Medpilot (advanced search; open search), all records published between January and December 2014

Date of search: 18 January 2015

| # | Search | Results |
| --- | --- | --- |
| 1 | exp basidiobolomycosis/ | 9 |
| 2 | exp basidiobolus/ | 7 |
| 3 | exp conidiobolomycosis/ | 3 |
| 4 | exp conidiobolus/ | 5 |
| 5 | exp entomophthoromycosis/ | 4 |
| 6 | exp entomophthora/ | 3 |
| 7 | exp facial swelling/ | 438 |
| 8 | exp phycomycosis/ | 1 |
| 9 | exp rhinoentomophthoromycosis/ | 0 |
| 10 | exp zygomycosis/ | 50 |
| 11 | Screening of reference lists of records identified in searches #1-#10 | 0 |
